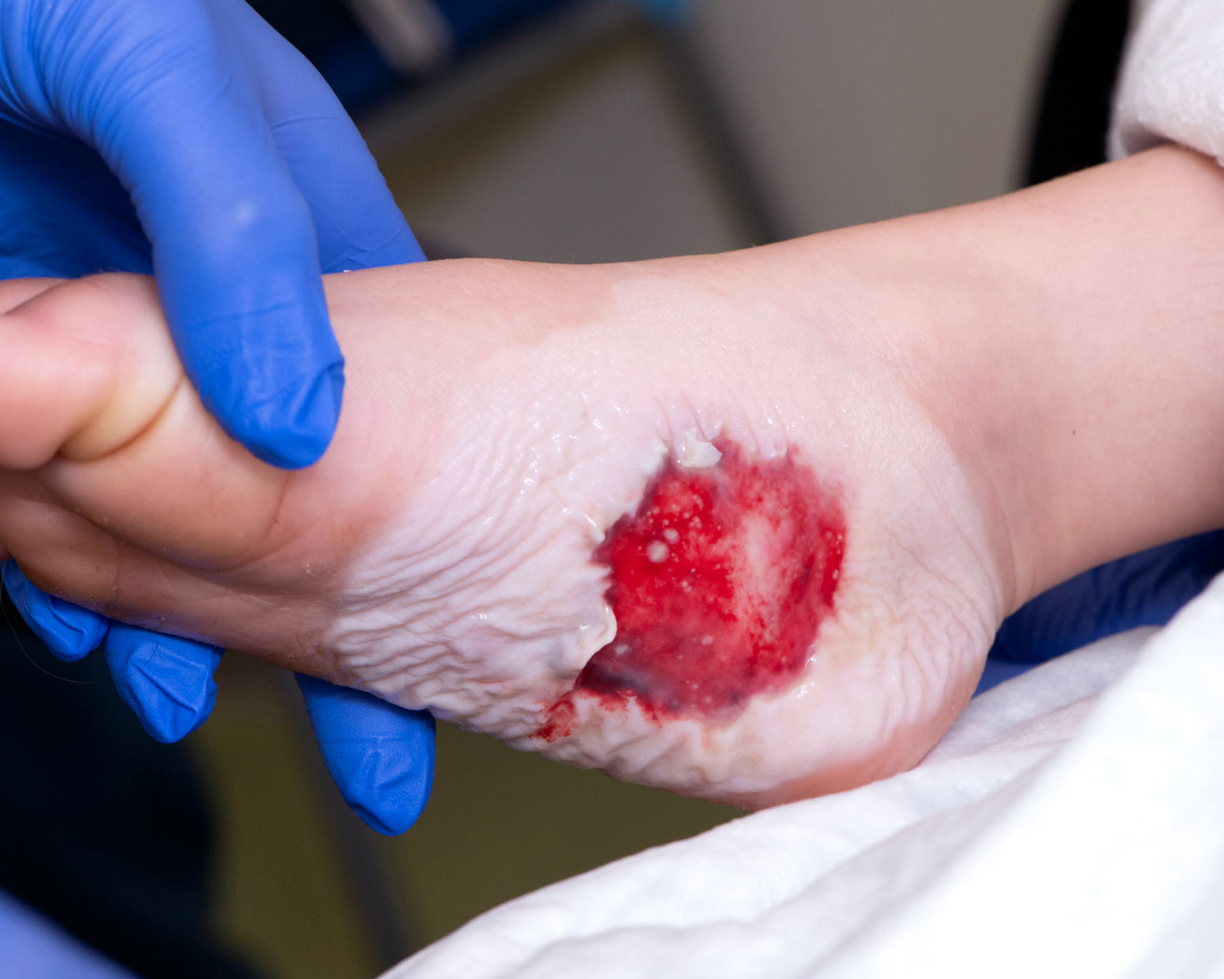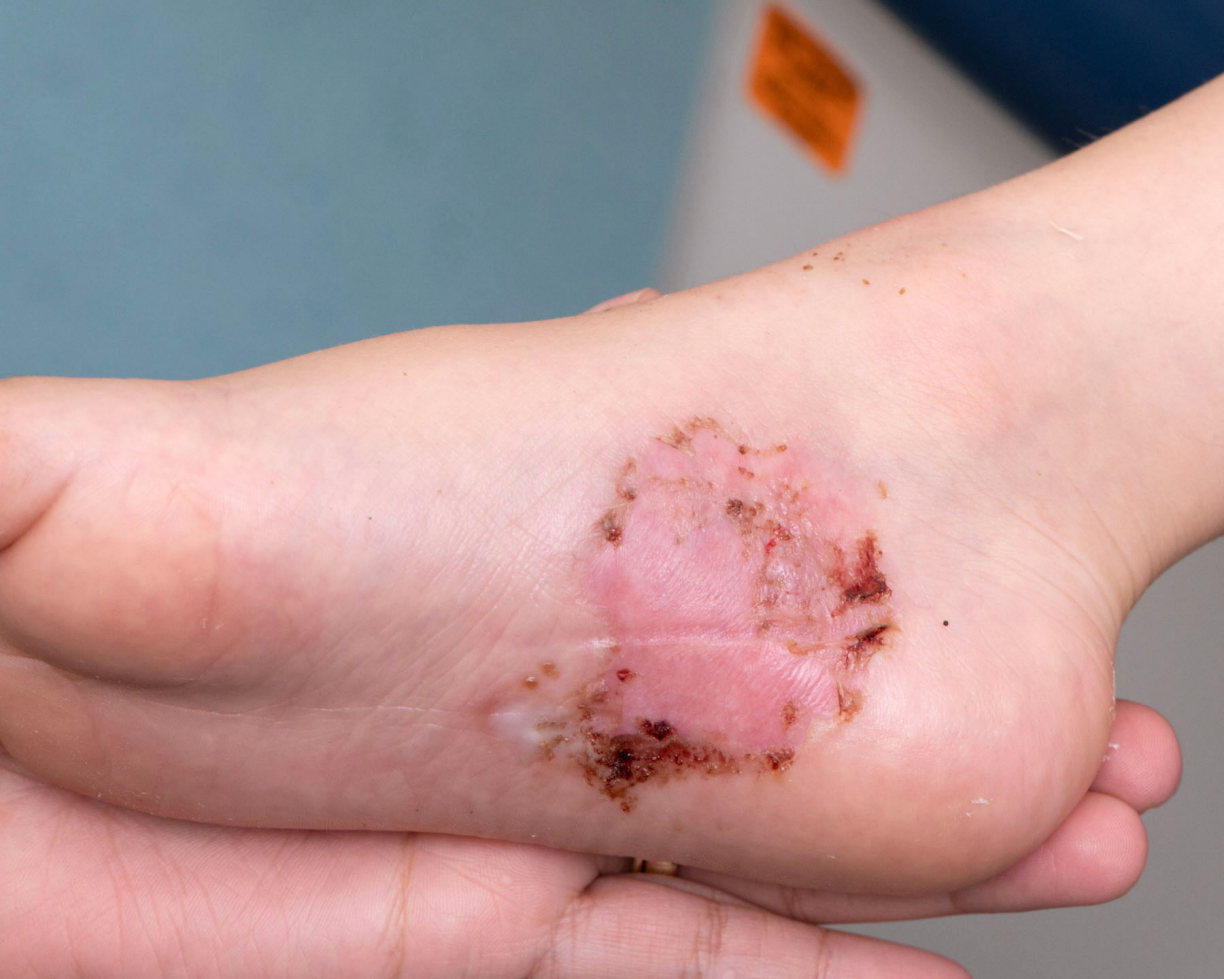# 841 Management of a Complex Avulsion-Type Injury Using Temporary Biosynthetic Wound Matrix

**DOI:** 10.1093/jbcr/iraf019.372

**Published:** 2025-04-01

**Authors:** Robyn Moore, Kerry Mikolaj

**Affiliations:** Children’s Hospital Colorado Burn Center; Children’s Hospital Colorado Burn Center

## Abstract

**Introduction:**

Avulsion-type injuries in areas of the body that experience high mechanical stress, such as the plantar surface of the foot, present significant challenges for wound management. Grafting in these frequently moving areas is often suboptimal in achieving adherence and healing, as they are at high risk of shear injury. A novel temporary biosynthetic wound matrix (TBWM) is a transparent, epidermal analogue designed to adhere and provide coverage to a wound bed quickly. This case study explores the use of TBWM in treating a 12-year-old male who sustained an avulsion injury to the medial arch and heel of the foot after entrapment in a bicycle spoke.

**Methods:**

The patient presented to the clinic two days post-injury with a mixed partial- and full-thickness wound accompanied by tissue loss (Figure 1). Blood flow to the wound was sufficient, except for central white clearing, suggesting compromised tissue in that region. The wound was prepped by soaking in an HOCL-based antimicrobial solution for 5 minutes. TBWM was then applied with a 1-2 cm margin around the wound, and 2-octyl cyanoacrylate was used to secure the TBWM. The treated area was covered with petroleum-based gauze Xeroform and wrapped in a soft cast.

**Results:**

At the two-week follow-up, the patient demonstrated satisfactory wound healing (Figure 3). TBWM’s adherent, transparent nature and perforations provided benefits in patient management. Specifically, the ability to bathe and apply adjunctive treatments without disturbing the wound dressing was highly advantageous as well as the ability visualize the healing wound bed at patient’s week one check-up. Overall, TBWM was easy to use and maintained adherence, even in a high-motion area. The patient and family expressed high satisfaction with the treatment, aside from a brief initial complaint of discomfort due to the dressing’s texture, which resolved after wound dressing.

**Conclusions:**

TBWM is an effective option for managing avulsion injuries in anatomically challenging locations where grafting is less favorable. This case highlights its convenience, ability to promote healing, and positive patient experience. These findings support the use of TBWM as a valuable skin substitute in complex wounds

**Applicability of Research to Practice:**

Managing complex wounds in areas subject to high mechanical stress, such as avulsion injuries on the plantar surface of the foot can be challenging. The novel use of a temporary biosynthetic wound matrix (TBWM) demonstrated effective wound coverage, adherence, and healing in a difficult-to-treat location. These findings suggest that TBWM can be a valuable alternative to traditional grafting methods, which often face challenges in burn care due to mobility and healing issues in similar high-motion areas. TBWM’s ease of application, durability, and positive patient outcomes highlight its potential in improving burn wound management.

**Funding for the Study:**

N/A